# Supplementary material for: Unveiling the Bioactive Potential of the Invasive Jellyfish Phyllorhiza punctata Through Integrative Transcriptomic and Proteomic Analyses
Source: Biomolecules. 2025 Aug 4;15(8):1121. doi: 10.3390/biom15081121 (PMC12383608; doi:10.3390/biom15081121)
Supplement: Supplementary file 1 [file biomolecules-15-01121-s001.zip › Suplementary Material/FileS3/PEPFOLD4/XP_065063041_11558/clusters.html]

 PEP-FOLD clusters


Download archive of all models  here   

|  |  |  |
| --- | --- | --- |
| # | file | sOPEP |
| # cluster 1 - Size: 60 |  |  |
|  | XP\_065063041\_11558-model1 | -17.5579 |
|  | XP\_065063041\_11558-model1.1 | -17.5042 |
|  | XP\_065063041\_11558-model1.2 | -17.5042 |
|  | XP\_065063041\_11558-model1.3 | -17.5042 |
|  | XP\_065063041\_11558-model1.4 | -17.5042 |
|  | XP\_065063041\_11558-model1.5 | -17.5042 |
|  | XP\_065063041\_11558-model1.6 | -17.5042 |
|  | XP\_065063041\_11558-model1.7 | -17.5042 |
|  | XP\_065063041\_11558-model1.8 | -17.5042 |
|  | XP\_065063041\_11558-model1.9 | -17.5042 |
|  | XP\_065063041\_11558-model1.10 | -17.5042 |
|  | XP\_065063041\_11558-model1.11 | -17.5042 |
|  | XP\_065063041\_11558-model1.12 | -17.5042 |
|  | XP\_065063041\_11558-model1.13 | -17.5042 |
|  | XP\_065063041\_11558-model1.14 | -17.4988 |
|  | XP\_065063041\_11558-model1.15 | -17.474 |
|  | XP\_065063041\_11558-model1.16 | -17.474 |
|  | XP\_065063041\_11558-model1.17 | -17.474 |
|  | XP\_065063041\_11558-model1.18 | -17.474 |
|  | XP\_065063041\_11558-model1.19 | -17.441 |
|  | XP\_065063041\_11558-model1.20 | -17.441 |
|  | XP\_065063041\_11558-model1.21 | -17.441 |
|  | XP\_065063041\_11558-model1.22 | -17.441 |
|  | XP\_065063041\_11558-model1.23 | -17.441 |
|  | XP\_065063041\_11558-model1.24 | -17.441 |
|  | XP\_065063041\_11558-model1.25 | -17.441 |
|  | XP\_065063041\_11558-model1.26 | -17.441 |
|  | XP\_065063041\_11558-model1.27 | -17.441 |
|  | XP\_065063041\_11558-model1.28 | -17.441 |
|  | XP\_065063041\_11558-model1.29 | -17.4001 |
|  | XP\_065063041\_11558-model1.30 | -17.4001 |
|  | XP\_065063041\_11558-model1.31 | -17.4001 |
|  | XP\_065063041\_11558-model1.32 | -17.4001 |
|  | XP\_065063041\_11558-model1.33 | -17.4001 |
|  | XP\_065063041\_11558-model1.34 | -17.4001 |
|  | XP\_065063041\_11558-model1.35 | -17.4001 |
|  | XP\_065063041\_11558-model1.36 | -17.4001 |
|  | XP\_065063041\_11558-model1.37 | -17.4001 |
|  | XP\_065063041\_11558-model1.38 | -17.4001 |
|  | XP\_065063041\_11558-model1.39 | -17.4001 |
|  | XP\_065063041\_11558-model1.40 | -17.3879 |
|  | XP\_065063041\_11558-model1.41 | -17.3879 |
|  | XP\_065063041\_11558-model1.42 | -17.3879 |
|  | XP\_065063041\_11558-model1.43 | -17.3879 |
|  | XP\_065063041\_11558-model1.44 | -17.3879 |
|  | XP\_065063041\_11558-model1.45 | -17.3879 |
|  | XP\_065063041\_11558-model1.46 | -17.3798 |
|  | XP\_065063041\_11558-model1.47 | -17.3622 |
|  | XP\_065063041\_11558-model1.48 | -17.3593 |
|  | XP\_065063041\_11558-model1.49 | -17.3593 |
|  | XP\_065063041\_11558-model1.50 | -17.3403 |
|  | XP\_065063041\_11558-model1.51 | -17.3305 |
|  | XP\_065063041\_11558-model1.52 | -17.3305 |
|  | XP\_065063041\_11558-model1.53 | -17.3235 |
|  | XP\_065063041\_11558-model1.54 | -17.2133 |
|  | XP\_065063041\_11558-model1.55 | -17.2133 |
|  | XP\_065063041\_11558-model1.56 | -17.2067 |
|  | XP\_065063041\_11558-model1.57 | -17.206 |
|  | XP\_065063041\_11558-model1.58 | -17.1206 |
|  | XP\_065063041\_11558-model1.59 | -16.9648 |
| # cluster 2 - Size: 26 |  |  |
|  | XP\_065063041\_11558-model2 | -13.28 |
|  | XP\_065063041\_11558-model2.1 | -13.2792 |
|  | XP\_065063041\_11558-model2.2 | -13.2574 |
|  | XP\_065063041\_11558-model2.3 | -13.2377 |
|  | XP\_065063041\_11558-model2.4 | -13.2377 |
|  | XP\_065063041\_11558-model2.5 | -13.2377 |
|  | XP\_065063041\_11558-model2.6 | -13.2377 |
|  | XP\_065063041\_11558-model2.7 | -13.2077 |
|  | XP\_065063041\_11558-model2.8 | -13.1894 |
|  | XP\_065063041\_11558-model2.9 | -13.1858 |
|  | XP\_065063041\_11558-model2.10 | -13.1858 |
|  | XP\_065063041\_11558-model2.11 | -13.1397 |
|  | XP\_065063041\_11558-model2.12 | -13.1397 |
|  | XP\_065063041\_11558-model2.13 | -13.1389 |
|  | XP\_065063041\_11558-model2.14 | -13.1389 |
|  | XP\_065063041\_11558-model2.15 | -13.1389 |
|  | XP\_065063041\_11558-model2.16 | -13.1345 |
|  | XP\_065063041\_11558-model2.17 | -13.1259 |
|  | XP\_065063041\_11558-model2.18 | -13.1232 |
|  | XP\_065063041\_11558-model2.19 | -13.0953 |
|  | XP\_065063041\_11558-model2.20 | -13.0894 |
|  | XP\_065063041\_11558-model2.21 | -13.0623 |
|  | XP\_065063041\_11558-model2.22 | -13.0622 |
|  | XP\_065063041\_11558-model2.23 | -12.9975 |
|  | XP\_065063041\_11558-model2.24 | -12.9881 |
|  | XP\_065063041\_11558-model2.25 | -12.9581 |
| # cluster 3 - Size: 14 |  |  |
|  | XP\_065063041\_11558-model3 | -13.1612 |
|  | XP\_065063041\_11558-model3.1 | -13.1612 |
|  | XP\_065063041\_11558-model3.2 | -13.1612 |
|  | XP\_065063041\_11558-model3.3 | -13.1568 |
|  | XP\_065063041\_11558-model3.4 | -13.1553 |
|  | XP\_065063041\_11558-model3.5 | -13.1481 |
|  | XP\_065063041\_11558-model3.6 | -13.1242 |
|  | XP\_065063041\_11558-model3.7 | -13.1107 |
|  | XP\_065063041\_11558-model3.8 | -13.1107 |
|  | XP\_065063041\_11558-model3.9 | -13.1038 |
|  | XP\_065063041\_11558-model3.10 | -13.0496 |
|  | XP\_065063041\_11558-model3.11 | -12.9972 |
|  | XP\_065063041\_11558-model3.12 | -12.9972 |
|  | XP\_065063041\_11558-model3.13 | -12.9327 |
|  |  |  |
